# Association of Republican partisanship with US citizens’ mobility during the first period of the COVID crisis

**DOI:** 10.1038/s41598-022-12790-5

**Published:** 2022-05-30

**Authors:** Guillaume Barbalat, Nicolas Franck

**Affiliations:** 1 Department of Population Health Sciences, Weill Cornell Medicine, 1300 York Ave, New York, NY 10065 USA; 2 Centre Ressource de Réhabilitation Psychosociale et de Remédiation Cognitive, Pôle Centre rive gauche, Hôpital Le Vinatier, UMR 5229, CNRS & Claude Bernard University Lyon 1, Université de Lyon, Lyon, France

**Keywords:** Environmental social sciences, Psychology and behaviour

## Abstract

While Republican states have been criticized for their limited efforts to contain the spread of COVID-19, it is important to consider that political orientation can modify human behaviour via complex effects that are still poorly understood. During the first period of the pandemic, we found that the association of Republican partisanship with US citizens' mobility varied depending on the nature of the exposure being considered. First, Republican partisanship was associated with increased mobility when the stringency of anti-COVID measures increased. Second, Republican partisanship was associated with decreased mobility when COVID-related deaths increased. Third, Republican partisanship was associated with increased mobility over time, i.e. as time went by, citizens living in Republican states were more mobile than those in Democratic states. These findings raise caution on any over-interpretation of the impact of polarization in US politics on COVID-related behaviour. They prompt consideration of persuasive tools that emphasize risk perception to promote social distancing in Republican states, rather than relying heavily on stringent anti-COVID interventions.

## Introduction

Republican states have been blamed for their relative leniency towards limiting the spread of SARS-COV-2^1,2^. This is especially true when considering efforts to increase social distancing and to restrict people’s mobility to places where the virus is mostly transmitted, e.g. transit stations, businesses, schools and universities^3–8^. In turn, these were thought to result in a higher overall prevalence of COVID cases and deaths in Republican than Democratic states^8–11^.

These differences in COVID-related infection and death rates have been accounted for by the lower strength of anti-COVID interventions observed overall in Republican states^9^. Yet, differences in political affiliation may also translate into differences in human mobility via more subtle, indirect effects. First, others have shown that citizens from Republican states were less compliant with stay-at-home orders compared with their Democratic counterparts^8,12^. This suggests that, even at similar levels of interventions, those with a Republican orientation would tend to be more lenient towards respecting anti-COVID measures. Second, changes in social distancing may also reflect individuals’ beliefs about disease risk, irrespective of government policies^13–16^. In other words, those who are more afraid of “catching the virus” would be more tempted to adopt self-protective behaviours (stay home rather than go out). Political orientation can greatly modify risk assessment, in particular citizens’ beliefs or level of concern about the virus^17^, which could have great consequences on their behaviour (including mobility) during the pandemic. Third, holding constant the strength of anti-COVID interventions and risk perception, citizens’ mobility could also vary as a function of time, for instance be attenuated or amplified as a result of varying intensity of enforcement, interventions on social media, nudging, or habituation, boredom and anger effects^18,19^. For instance, over time citizens have received various messages from political parties, accurate or not, about the need to distance and the severity of the disease^20,21^, and more recently about vaccination risks and effectiveness.

Little is known about these *moderating* effects of political orientation on citizens’ behaviour in response to the COVID crisis. Their investigation would however be of paramount importance to better understand the natural tendencies and fine-grained mechanisms underlying the influence of political affiliation on people’s behaviour, and further, how to counteract them when they result in significant health and social burden. In the current study, we sought to determine how Republican partisanship affected US citizens’ behaviour during the first period of the pandemic, via its effect on citizens’ compliance with anti-COVID measures, on their perception of COVID-related risks, as well as over time. We chose mobility to transit stations as our main outcome variable since that measure would theoretically be closely linked to a higher spread of the virus^22^. We ran sensitivity analyses on other types of mobility and in general obtained similar results. As proposed by others, we used the local cumulative number of COVID deaths and cases as proxies for informational inputs about general perception of COVID risks during the pandemic^19^. Finally, we chose to circumscribe our analysis to the beginning of the pandemic (from Mid-February to end of May 2020) as we did not want the US presidential elections campaign to amplify the bias inherent to political polarization on citizens’ behavioural response.

## Methods

### Data

#### Outcome variable (mobility to transit stations)

We used the *Google COVID-19 Community Mobility Reports* database to extract state-wise daily percentage change in mobility to transit stations^23^. This database has been used extensively to assess various interventions during the pandemic. It provides longitudinal data for six particular categories of mobility data: (1) transit stations; (2) retail and recreation places; (3) workplaces; (4) groceries and pharmacies; (5) residential places; (6) parks. Observations are based on cellular phone, laptop and tablet signals. Specifically, the data shows how visits and time spent in the above places change compared to so-called “baseline days”. For each day of the week, the baseline day is the median value from the 5‑week period Jan 3–Feb 6 2020 (i.e. before the pandemic hit the US, hence before restrictions on movement) for a specific location and a specific mobility category. For each day, an integer value gives the percent change in mobility (positive or negative) compared to that same day during that 5-week period (Jan 3–Feb 6).

In the current study, we chose mobility to transit stations as our main outcome of interest and used other measures in our sensitivity analysis^24^. We chose to restrict our time series to the period from February the 15th 2020 (start date of the database) till May the 31th 2020. The latter is often taken as the end of the first wave of the pandemic. In total, our time series amounts to 107 days. Overall, our data comprised N = 51 states × 107 days = 5457 observations. There were no missing values.

#### Political orientation

Each state’s political orientation was defined as each state trifecta as of 2020 (Democratic vs. divided vs. Republican) and retrieved from the ballotpedia website^25^. A state government trifecta occurs when either the Republican or the Democratic party holds the governorship and a majority in both chambers of a state's legislature. States where neither party holds trifecta control are reported to be “divided”. In the Supplementary Materials 1, we provide a list and a map of US states according to their political affiliation.

#### Stringency of anti-COVID measures

For each state and each day, a measure of the stringency of anti-COVID interventions was extracted from the Oxford COVID-19 Government Response Tracker (OxCGRT)^26^. In short, the OxCGRT provides a measure of stringency on a scale from 0 to 100, publicly available on GitHub^27^. This measure is based on a summary of seven indicators on policies regarding social isolation and confinement, including school, workplace, and public transport closures, public events cancellations, stay at home requirements, and restrictions on gatherings and internal movement. Data is collected from publicly available sources such as news articles and government press releases and briefings. Each indicator measures the stringency of each policy or intervention on an ordinal scale of severity or intensity (from no measures taken to simple recommendations and implementation of the policy). Note that OxCGRT measures for US states do not include federal policies that apply to the country as a whole (e.g., international travel bans).

We reasoned that the effect of anti-COVID measures would be expected on citizens’ mobility on the day they were introduced, as in^28^, therefore we did not include any lags in the “Stringency” time series.

#### Cumulative number of COVID deaths

We used the daily cumulative number of COVID deaths in each state as a reflection of the amount of risks due to COVID perceived daily by citizens in each state^19^. Indeed, we reasoned that a great marginal increase in the number of COVID-related deaths would inform citizens that the current status of the pandemic may be dangerous. And in turn, a large (negative) effect of the number of COVID deaths on mobility would suggest that citizens take great precautions against the virus by not going out. For each state and each day, the cumulative number of confirmed COVID-related deaths was extracted from the Oxford COVID-19 Government Response Tracker, and lagged by one day^27^. Note that for the purpose of our research question, we indeed reasoned that an optimal way to model the impact of COVID deaths on citizens' mobility would be to use the raw number of deaths rather than rates. This is because our hypothesis relies on the impact of COVID deaths on mobility via people’s perceptions of the danger to go out due to an increased number of COVID deaths. In that sense, we hypothesized that a raw number would be more meaningful to people than a (relatively small) rate.

#### Temperature

We included a climate variable in our statistical model as we reasoned that climate might confound the relationship between COVID deaths and mobility. We retrieved the mean temperature (in degrees Fahrenheit) of each state’s capital city for each day of our time series from the *wunderground* website^29^.

### Analysis

#### Main analysis

We specified multivariate linear models using directed acyclic graphs to include variables that appeared to determine mobility to transit stations (Supplementary Materials 2). Specifically, we fit our time series of the mobility to transit stations according to the following linear equation:

$$\begin{aligned} Mobility_{sd } = & \beta_{0} + \beta_{1} Stringency_{sd} + \beta_{2} Stringency_{sd} *Republican_{s} \\ & + \beta_{3} Deaths_{sd} + \beta_{4} Deaths_{sd} *Republican_{s} \\ & + \beta_{5} Temp_{sd} + \beta_{6} Temp^{2}_{sd} \\ & + \beta_{7} Trend_{d} *Republican_{s} \\ & + \theta_{s} + \delta_{d} + \epsilon_{sd} \\ \end{aligned}$$

where:

- $Mobility_{sd }$ is the percent signal change from baseline in mobility to transit station, for state $s$ at day $d$;
- $Stringency_{sd}$ is a measure of the stringency of anti-COVID interventions, for state $s$ at day $d$*;*
- $Republican_{s}$ is an indicator of Republican partisanship for each state $s$. For the purpose of our main analysis, we define a crude index of Republican partisanship according to each state’s trifecta, where Democratic states would be affiliated the value of 0, divided states the value of 1, and Republican states the value of 2;
- $Death{s}_{sd}$ is the number of COVID deaths (cumulative) for state $s$ at day $d$;
- $Tem{p}_{sd}$ and $Tem{{p}^{2}}_{sd}$ are measures of the mean temperature of each state $s$ capital city at day $d$ (raw and squared terms, respectively);
- $Tren{d}_{d}$ indicates temporal count, taking the value $d$ at day $d$*.* Note that we also tested a model including a non-linear, quadratic temporal trend, which we subsequently decided to remove as it was non-significant;
- ${\beta }_{i}$ indicates the parameter estimate of the respective regressor $i$. We were specifically interested in ${\beta }_{2}$ and ${\beta }_{4}$, which represent the effect of anti-COVID stringency measures and cumulative COVID Deaths on citizens’ mobility moderated by Republican partisanship, respectively. We were also interested in ${\beta }_{7}$, which represents the effect of Republican partisanship on mobility over time;
- ${\theta }_{s}$ represent states fixed effects for each state $s$. States fixed effects are “omitted” and unobserved state-level time-invariant factors that distinguish US states and that could bias our statistical estimates of interest. These would be confounders in the relationship between treatments and outcome, i.e. factors that may influence anti-COVID measures and/or the cumulative number of COVID deaths, as well as citizens' mobility. Such factors might be (but are not restricted to): population-level risk preferences^30^, general level of trust to politicians and healthcare systems^31^, culture, ethnicity and religion, population density, percentage of urban population, share of population aged 65 or older, average household size, unemployment rate, income per capita and income inequality^32–34^, percentage of the population with risk factors for COVID-19 (such as obesity or cardio-vascular disease), state’s physician rate;
- ${\delta }_{d}$ represent days fixed effect for each day $d$. Days fixed effects model time-varying state-invariant factors that may confound our main relationships of interest. In short, these would be represented by nationwide daily variability in movement, for instance due to national holidays, weekend days, vacation periods, economic downturns, or nationwide weather patterns;
- ${\epsilon }_{sd}$ is the model residual for state $s$ at day $d$.

Because policy stringency and the number of COVID deaths likely correlate with the time variable, the presence of multicollinearity among variables included in the model was investigated. The variance inflation factor was below 7.5 for each regressor, and below 5 for our three regressors of interest. When we further scaled the temperature variable (linear and quadratic terms), the variance inflation factor was below 5 for each regressor, without any change in the mean coefficient value for each parameter of interest (${\beta }_{2}$,${\beta }_{4}$ and ${\beta }_{7}$).

Cluster robust standard errors were estimated to account for the correlation of data within each state. Note that we did not include a main effect of Republican partisanship, nor a single linear temporal trend in our statistical model as these would be collinear with states and days fixed effects, respectively.

#### Event-studies

We performed additional analysis where variables *Stringency*, *Deaths* and *Trend* were discretized. Specifically, *Stringency*, *Deaths* and *Trend* were discretized into 10 categorical bins, such that each bin contained enough data to perform additional regression analyses (see Supplementary Materials 4 for a table summarizing the number of observations contained in each bin for each political group). Doing so, the chances of data sparsity were reduced, which improves the interpretability of our results. Another reason why we performed these additional analyses is that they provide better insights on the effect modification of *Stringency*, *Deaths* and *Trend* by Republican partisanship, because discretization allows better visualization of subtle non-linear effects.

We specified the following linear equation:

$$\begin{aligned} Mobility_{sd } = & \beta_{0} + \beta_{1} Stringency_{sd} + \mathop \sum \limits_{i = 1}^{10} \alpha_{i} \left( {BinStringency_{sdi} *Republican_{s} } \right) \\ & + \beta_{3} Deaths_{sd} + \mathop \sum \limits_{j = 1}^{10} \gamma_{j} \left( {BinDeaths_{sdj} *Republican_{s} } \right) \\ & + \beta_{5} Temp_{sd} + \beta_{6} Temp^{2}_{sd} \\ & + \mathop \sum \limits_{k = 1}^{10} \lambda_{k } \left( {BinTrend_{sdk} *Republican_{s} } \right) \\ & + \theta_{s} + \delta_{d} + \epsilon_{sd} \\ \end{aligned}$$

where $BinStringenc{y}_{sdi}$, $BinDeath{s}_{sdj}$ and $BinTren{d}_{sdk}$ take the value of 1 if $BinStringenc{y}_{sdi}$, $BinDeath{s}_{sdj}$ and $BinTren{d}_{sdk}$ correspond to the value of $i,j,k$, respectively.

${\alpha }_{1}, {\gamma }_{1 }, {\lambda }_{1}$ are the reference coefficients and are set to 0.

${\alpha }_{i}, {\gamma }_{j }, {\lambda }_{k}$ therefore indicates the effects of Republican partisanship on mobility at each bin $i,j,k$ comparatively to that at bins $i=1, j=1, and \,k=1$ for Stringency, Deaths and Trend, respectively.

We then reported so-called “event-study plots” to illustrate the effect of each discretized variable, modified by Republican partisanship, on the outcome.

#### Sensitivity analysis

We ran the following sensitivity analysis to check that subtle changes in measurement would not invalidate our conclusions.

##### Outcome variable

Here, we substituted mobility to transit stations to that of other places where signals were recorded in the Google COVID-19 Community Mobility Reports database: retail and recreation places, workplaces, groceries and pharmacies, residential places, and parks.

We hypothesized that our estimates of interest would not substantially change as compared to those obtained with mobility to transit stations when outcome variables were mobility to retail and recreation places, workplaces, and groceries and pharmacies. For residential places, we hypothesized that estimates would be of an opposite sign than those obtained with transit stations. Finally, we hypothesized that estimates would be non-significant for mobility to parks, as we reasoned that these would be much less impacted by subtle exposure changes.

##### Measurement of Republican partisanship

Here, we changed our strategy for measurement of Republican partisanship. Using data from the Pew Research Center, we differentiated states in terms of their percentage of voters for the Republican candidate at the 2016 presidential elections (former President Trump), rather than a rough differentiation of their political group as we did in the main analysis (Democratic vs. divided vs. Republican states) ^35^.

##### Measurement of risk perception

We re-ran our main analysis using cumulative COVID cases instead of cumulative COVID deaths as a measure of perception of COVID risk.

##### Accounts of temporality

We first tested a model taking into account a specific correlation structure of the error term, as sequences of non-seasonal Autoregressive Moving Average (ARMA) of daily values for each state, with *p* = 1 lag.

Second, to test whether our results were robust to a longer period of analysis, we reproduced our event-study plots (aming to illustrate the effect of each variable of interest, modified by Republican partisanship) over a longer period of analysis (till the end of February 2021 rather than end of May 2020).

Analyses were performed R version 4.0 and packages *daggity*, *fixest* and *nlme*.

## Results

Our multivariate time series analysis aimed to linearly fit, among US states, daily mobility to transit stations (obtained from the publicly available Google COVID-19 Community Mobility Reports database) to anti-COVID stringency interventions (the so-called “Stringency” term in our statistical model), number of COVID deaths (“Deaths” term), as well as their interaction with an index of Republican partisanship (“Republican ” term). Republican partisanship was based on each state’s government trifecta, and was defined as a linear increase from Democratic trifectas to divided governments and further, to Republican trifectas. We were also interested in the effect of Republican partisanship on mobility over time (“Trend” term). Note that our model also included an index of climate (daily temperature in each state—raw and squared terms), as well as states and days fixed effects. The latter allowed to remove the variance related to state- and time-wise factors that could have had an impact on mobility to transit stations and that could have confounded the effect of party affiliation (e.g. unemployment rate, ethnicity, population density, national holidays etc.…).

### Data description

**Figure 1:**
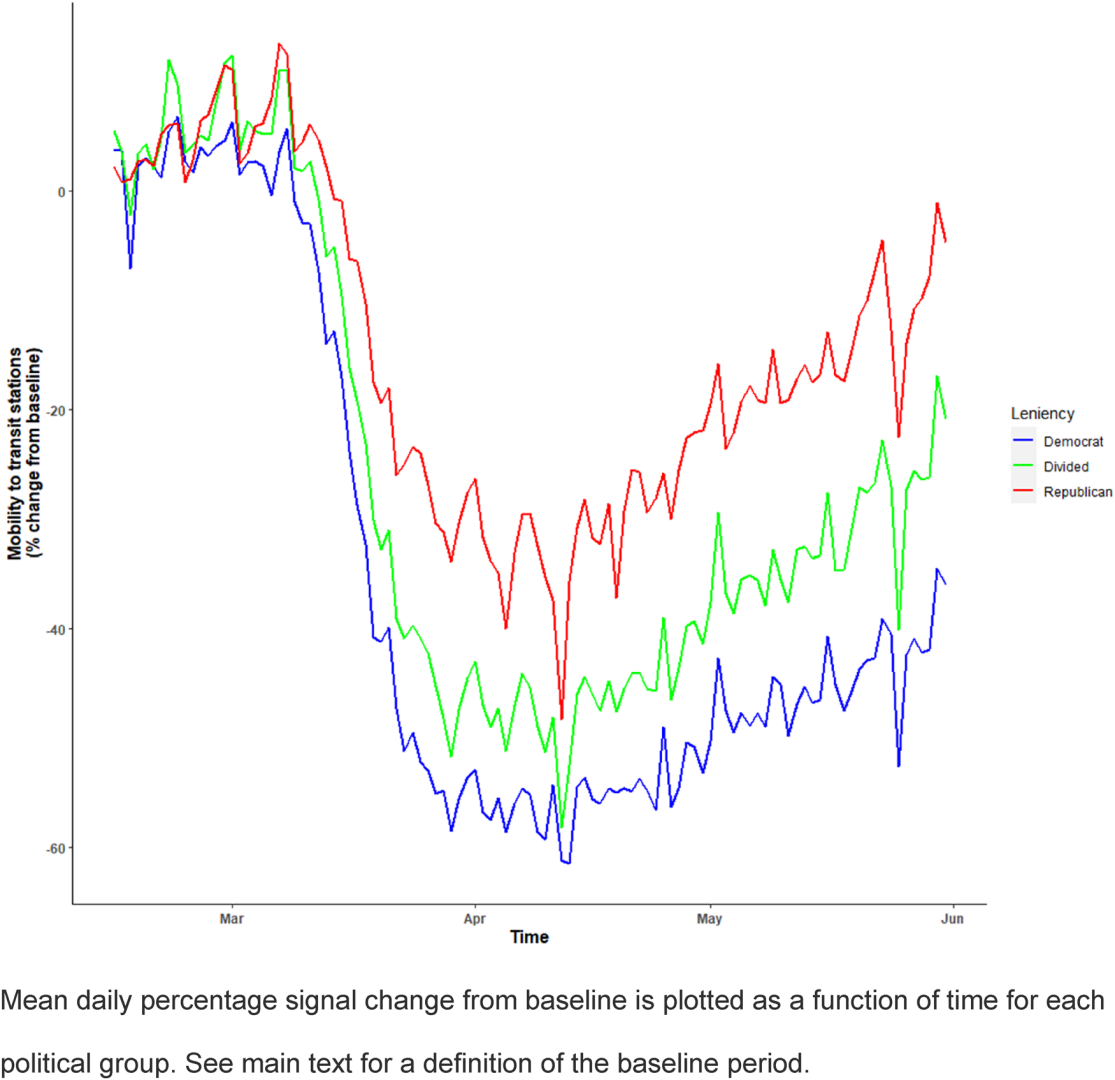
US citizens’ mobility to transit stations for each political group during the first period of the pandemic (Feb 15 to May 31 2020).

Figure 1 provides a plot of the mean trend for the outcome variable (mobility to transit stations) for each political group (also see Supplementary Materials 3. A&B). Mobility to transit stations started to decrease after the first week of March and started to rebound after the first week of April. Overall, Republican states presented with a higher level of mobility than divided and Democratic states.

**Figure 2:**
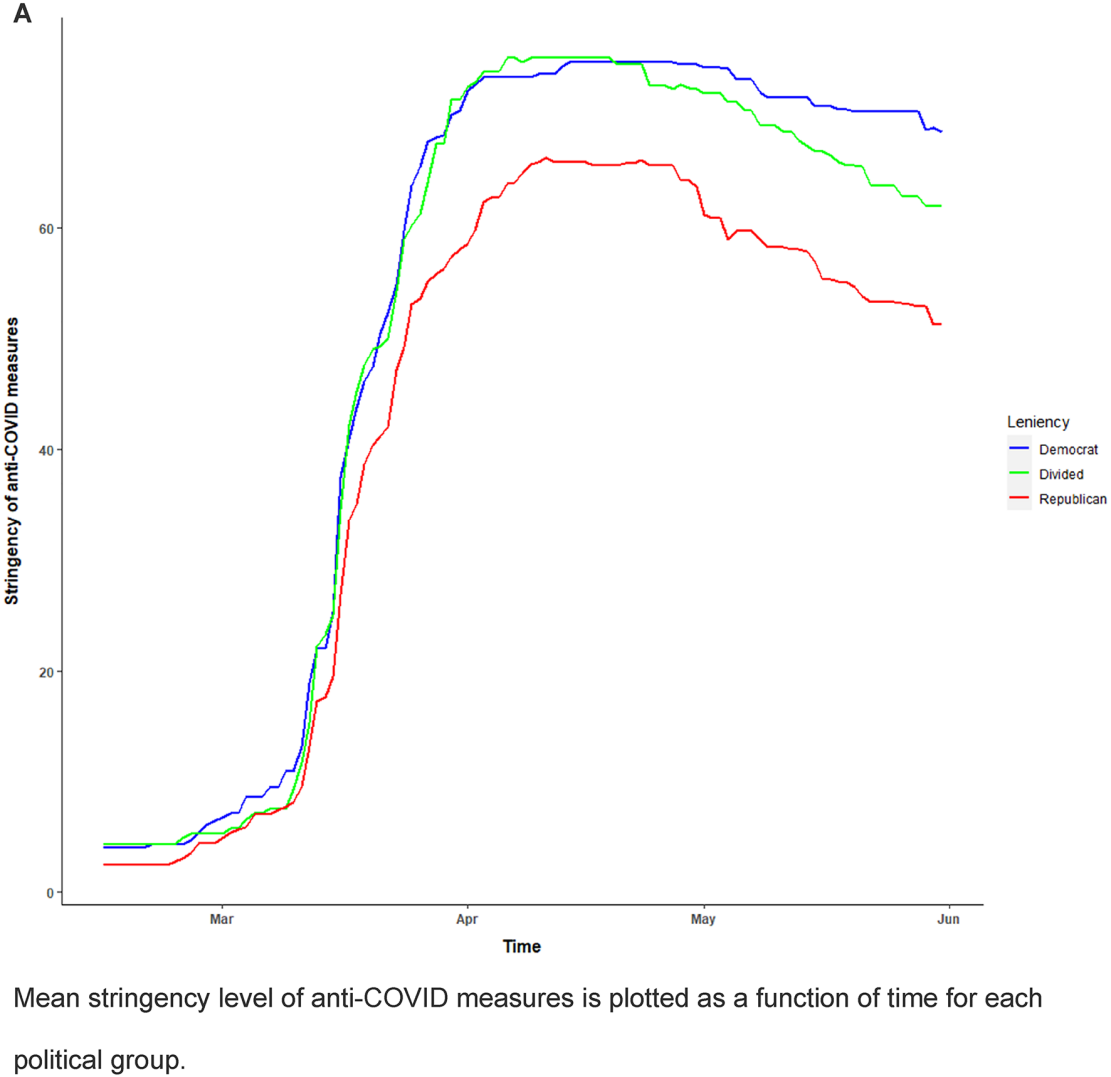

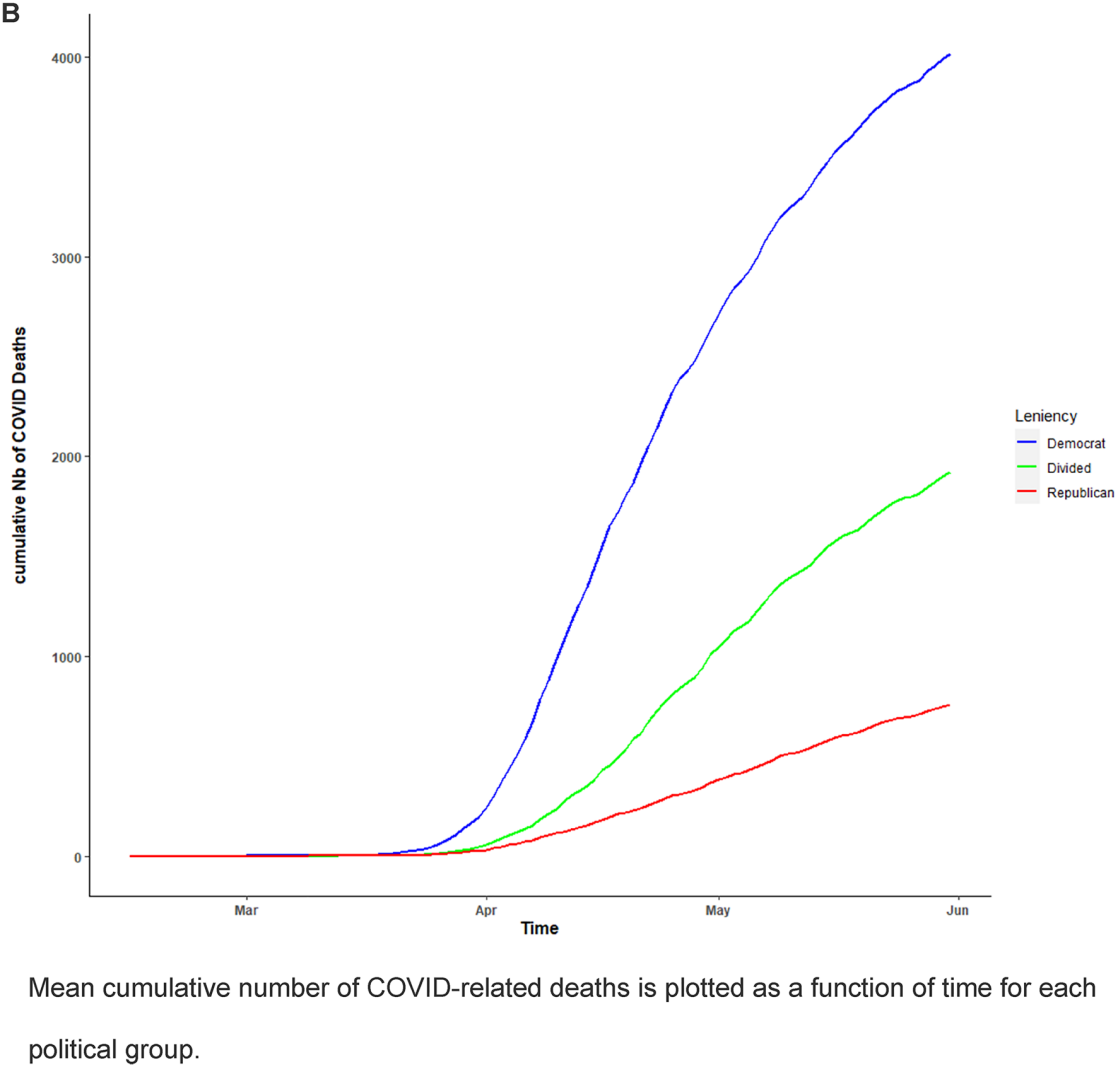
Stringency of anti-COVID measures (**A**) and Number of COVID-related deaths (**B**) for each political group during the first period of the pandemic (Feb 15 to May 31 2020).

Figure 2 provides a plot of the mean trends for the stringency of anti-COVID interventions and the cumulative number of COVID-related deaths for each political group (also see Supplementary Materials 3. C-F). Overall, Republican states underwent a lower strength of anti-COVID measures, and presented with a lower number of COVID deaths than Democratic and divided states. The latter is in line with a previous report showing that rates of COVID cases and deaths in Republican states started to exceed those in Democratic states from June 2020 onwards^11^.

### Main results

Table 1 provides parameter estimates for each term of our statistical model. As expected, there was a main effect of stringency of anti-COVID measures as well as number of COVID deaths on mobility to transit stations, such that more stringent anti-COVID interventions and a higher number of COVID deaths were related to a decrease in mobility to transit stations (*p*’s < 0.0073). An increase in temperature was also related to a significant increase in mobility (*p* = 0.0475), but the temperature square term did not have any significant effect (*p* = 0.1952).

**Table 1:** Parameter estimates, standard errors (SE) and p values from our main model (with mobility to transit stations as the dependent variable).

	Mobility to transit stations		
Variable	Estimate	SE	*p* value
Stringency	− 0.2126	0.0673	**0.0027**
COVID deaths	− 0.0003	0.0001	**0.0073**
Temp	0.4187	0.2060	**0.0475**
Temp^2	− 0.0027	0.0020	0.1952
Stringency × Republican	0.0810	0.0282	**0.0060**
COVID Deaths × Republican	− 0.0031	0.0007	**0.0000**
Temporal linear trend × Republican	0.1076	0.0201	**0.0000**

Significant values are in [bold].

Interactions between our index of Republican partisanship with (1) stringency of anti-COVID measures, (2) number of COVID deaths and (3) linear temporal trend were all significant (*p*’s < 0.006). Specifically, the effect of stringency on mobility was moderated by Republican partisanship, such that Republican partisanship was associated with increased mobility at higher stringency levels (main effect of Stringency: b = − 0.2126, *p* = 0.0027; Stringency × Republican interaction: b = 0.0810, *p* = 0.006). All other parameters being kept constant, an increase of 50 units on the Stringency scale was associated with a decrease of 10.5% in the percentage signal change in mobility to transit stations. Republican partisanship however increased this percentage by about 4%. The effect of COVID deaths was also modified by Republican partisanship, such that Republican partisanship was associated with decreased mobility when the number of COVID deaths increased (main effect of Deaths: b = − 0.0003, *p* = 0.0073; Deaths x Republican interaction: b = − 0.0031, *p* < 0.0001). All other parameters being kept constant, an increase in the cumulative number of COVID-related deaths by 10,000 was associated with a decrease of about 3% in the percentage signal change in mobility to transit stations. Republican partisanship further decreased this percentage by 31%. Finally, Republican partisanship was associated with increased mobility as a function of time (Trend x Republican interaction: b = 0.1076, *p* < 0.0001). All other parameters being kept constant, in 10 additional days, Republican partisanship increased the percentage signal change in mobility to transit stations by an additional 1.1%.

**Figure 3:**
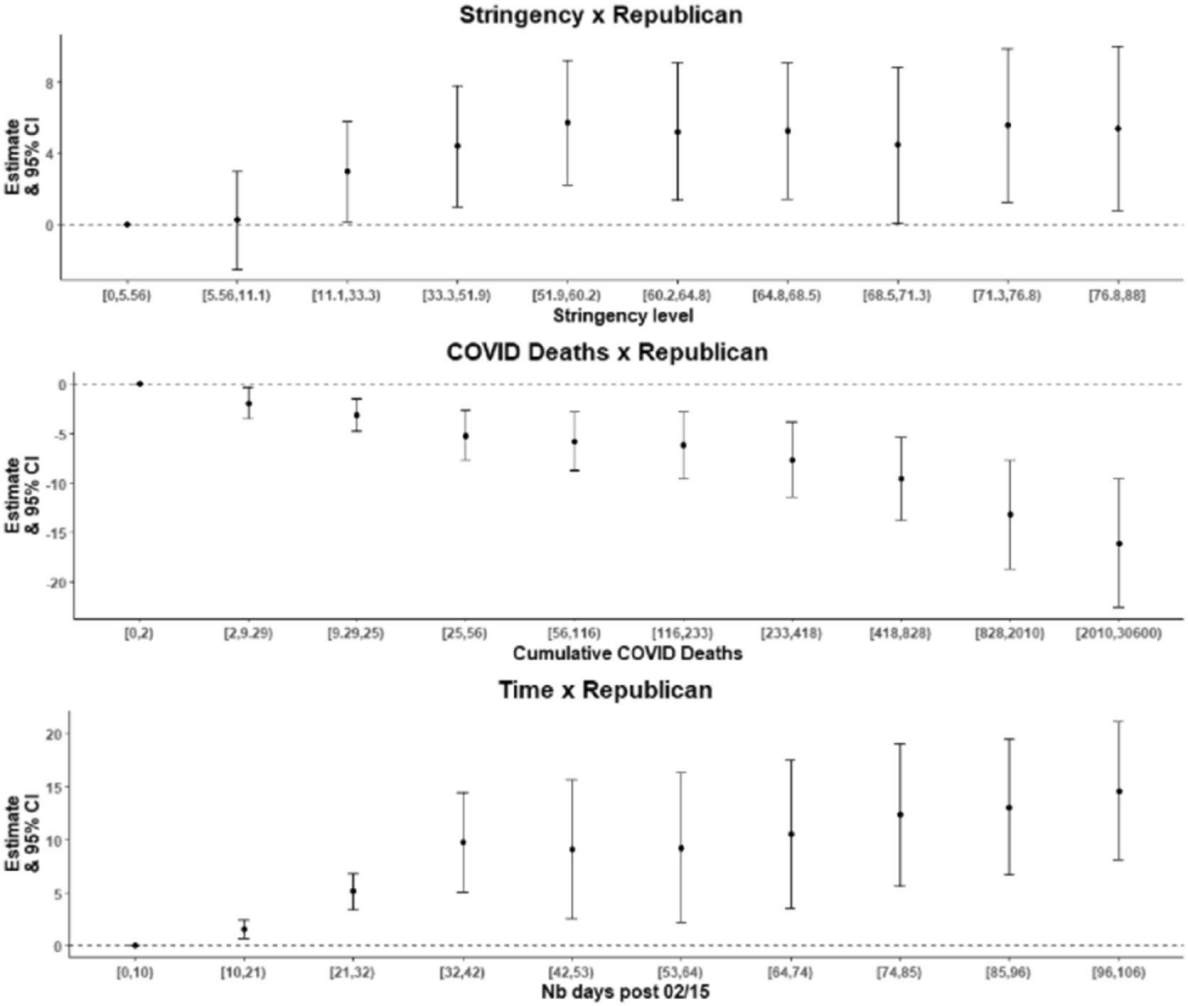
Event-study plots representing the effect of stringency of anti-COVID measures, number of COVID deaths and time on mobility to transit stations, modified by Republican partisanship. In each panel, the reference represents the effect of Republican partisanship on mobility at the lowest value of the variable represented on the x axis (i.e. the lowest stringency level, the lowest number of COVID-related deaths, and the lowest number of days after Feb. 15 2020—the beginning of the time series). At the reference point, the value of the corresponding parameter estimate is of 0. Other estimates have to be interpreted relative to the reference point. For instance, compared to the reference point, increasing the stringency of anti-COVID measures is associated with an increased effect of Republican partisanship on mobility to transit stations.

Figure 3 provides event-study plots for each of our three effects of interest (Stringency × Republican, Deaths × Republican, Trend × Republican). In general, our event-studies confirmed the significance of these three interaction terms obtained from our main analysis.

### Robustness checks

Our sensitivity analysis revealed that our results were in general robust to changes in our outcome variable (using various measures of mobility), measurement of Republican partisanship (using the percentage of citizens voting for the Republican candidate at the 2016 presidential elections), measurement of risk perception (using number of COVID cases rather than deaths), and accounts of temporality (taking into account the temporal correlation structure of the error term and increasing the duration of our time series) (Supplementary Materials 5).

In terms of our sensitivity analysis on outcomes, we retrieved relatively similar coefficients for Stringency × Republican, Deaths × Republican and Trend × Republican when using mobility to retail and recreation places, workplaces, or groceries and pharmacies as our dependent variable. Compared to mobility to transit stations, specifying models with mobility to residential places as our outcome demonstrated effects of an opposite sign. Finally, mobility to parks did not demonstrate any significant effect of Stringency × Republican, nor Deaths × Republican.

## Discussion

Our study provides an analysis of the association between Republican partisanship and US citizens' mobility during the first period of the COVID-19 pandemic. Using mobility to transit stations as our dependent variable, we found that Republican partisanship was associated with increased mobility when the stringency of anti-COVID measures increased, but decreased mobility when the number of COVID deaths increased. Over time, we observed a positive effect of Republican partisanship on mobility: as time went by, citizens living in Republican states were more mobile than those in Democratic states. We retrieved relatively similar results when running sensitivity analysis on outcomes, *modulo* subtle differences that could be due to our use of a rather general measure for the strength of anti-COVID interventions.

Our study adds to the growing body of literature showing that political polarization impacts health-related behaviours, from enrollment in health care insurance to perceptions about the safety of vaccines^7,8,12,17,36–41^. Yet, our design also allowed us to improve our understanding of the effect of political orientation on citizens’ response to the pandemic at a fine-grained level. We first showed that, in terms of mobility to transit stations, Republican partisanship was associated with a lower compliance with anti-COVID interventions. This confirms previous findings that alignment with political ideas could bias the formation of opinions and behaviours, even when these ideas are not substantive^39^, and that party endorsement mitigates adherence to evidence-based policy directives^42^. More specifically, psychological underpinnings typically related to Republican partisanship might be inherently unaligned with highly constraining health-related policies, even though they could benefit the whole community^43^. For instance, while being usually referred to as people of honor and duty, that respect authority, religion and traditions^44,45^, Republican citizens demonstrate significant antisocial traits^46–48^ and, when reflecting upon public health-related matters, show a low level of trust in their governments and healthcare providers^19,30,31,49,50^. Likewise, from the perspective of cultural norms, Republican citizens demonstrate less communion traits than their Democratic counterparts, and more individual, agency traits^51^. Overall, these psychological characteristics might have biased citizens from Republican states towards more reluctance to comply with constraining, “high-cost” anti-COVID measures taken by their local governments.

Second, in line with previous theories and research, we reasoned that, irrespective of government policies, learning the number of local COVID deaths would impact self-protective behaviours, because of a heightened perception of risk to catch the virus, and/or of the disease severity^15^. Confirming these predictions, we found an association between a greater number of COVID deaths and a decreased likelihood of going out to transit stations. Yet, we also demonstrated variations in such risk assessments across political groups, with individuals from Republican states being overall more cautious vis-a-vis the virus. Given previous research, we hypothesize that this over-cautiousness associated with Republican partisanship might relate to: (1) a different age structure, i.e. a higher rate of older aged people who would be more risk-averse towards the virus^52^; (2) a specific cognitive profile, with less extraversion and risk-taking behaviours associated with Republican partisanship^31,32^; and/or (3) a heightened response to fearful stimuli in Republican citizens^53–55^. Further research should explore how each of these factors may contribute to the perception of COVID-related risks in citizens that are differently affiliated to political parties.

Our third finding pertains to the significant difference in the temporal variation of mobility to transit stations across political groups, with an increased temporal trend in mobility associated with Republican partisanship^8,41^. One potential explanation for such a temporal pattern is that the economic burden that mobility restrictions increasingly impose on individuals would be differentially appraised, and palliated for, in Republican vs. Democratic states. As an example, Republican citizens and governors demonstrated more reluctance to get federal unemployment aid^56^, which, in turn, could have resulted in increased mobility over time related to job hunting. A second hypothesis is that protective, yet constraining behaviours that relate to low levels of mobility may be differently integrated to social norms^57^ in Democratic vs. Republican states. Indeed, some have shown that Democratic citizens were in general more motivated to socially distance^58^, while others, more likely to be Republicans, would demonstrate a heightened degree of so-called “pandemic fatigue”^59–61^. Thirdly, these temporal variations may have resulted from specific events, a.k.a. discontinuities in the time series. Typically, political speeches or interventions on social media, such as statements from President Trump that “America wasn’t built to be shut down”, or that “We cannot let the cure be worse than the problem itself”, may have influenced citizens from Republican states more than those from Democratic states, which in turn would have resulted in the former being less inclined to socially distance than the latter^20,21,62^.

Our study has some limitations. First, when making causal inference, one should recall that the interpretation of our results should always remain at the state level in order to avoid the so-called “ecological inference fallacy”. Lower-level analyses (e.g. at the county level) may be considered more accurate, yet at the cost of an increased complexity of both the study design and the statistical model (e.g. with an increased number of higher-order interactions). In that sense, our results could be regarded as a first step to further inform more complex, lower-level investigations.

Second, the Google COVID-19 Community Mobility Reports database provides measures on mobility based on cellular phone signals, which would not capture mobility of people who do not have cell phones or have them turned off. In addition, this “measurement error” could also be correlated with certain states characteristics, their political orientation, the number of COVID deaths, and the stringency of anti-COVID measures. Finally, using the number of COVID deaths (or cases) as a proxy for citizens’ risk perception may not have accounted for their level of interest in the pandemic^6,43,62^.

Third, in the current study, we made use of states and days fixed effects to control for any time-invariant state-level and time-varying unit-invariant unmeasured confounders, respectively. However, in this observational study, omitted variable bias might still exist, because some of the unobserved confounders taken into account in our fixed effects model might interact with our variables of interest and modify our outcome. Future studies should investigate how parameter estimates related to political orientation are themselves modified across different types of socio-demographic strata.

Fourth, we acknowledge that our statistical model might be suboptimal. Indeed, at this point, one lacks strong knowledge to properly model longitudinal effects from outcomes (citizens’ mobility) at time t to exposures (anti-COVID measures and COVID-related deaths) at time t + j, as well as from exposures at time t to other exposures at time t + k. This is because there is a high degree of uncertainty in the lags j and k, which in turn would typically result in some form of simultaneity bias.

Fifth, in our event-studies, while we were careful that each stratum of covariates contained a substantial number of data for each of the three political categories (to avoid data sparsity), one could still argue that our analysis would be biased by the fact that Democratic states presented with a higher strength of anti-COVID interventions and a greater number of COVID deaths than Republican states. However, we found a significant effect of Republican partisanship at low stringency levels and at low levels of COVID deaths, which suggests that our reported estimates are likely not fallacious.

In short, our parameter estimates of interest should be interpreted as representing estimates of adjusted associations rather than causal estimates per se. With these precautions in mind, we found Republican partisanship to be associated with increased mobility when the stringency of anti-COVID measures increased, as well as over time. In contrast, Republican partisanship was associated with decreased mobility when COVID-related deaths increased. These results raise caution on any simplistic over-interpretation of the impact of polarization in US politics on citizens’ behaviour, and more particularly towards over-blaming and stigmatizing Republican states. They also suggest that specific measures could be implemented in Republican states to ensure the lowest possible rates of infection. Relying too much on constraining, “high-costs” measures that are tough to be scrupulously respected, such as business, university and school closures, or stay-at-home orders, might not represent an optimal solution in Republican states. Rather, citizens living in Republican states might be more easily persuaded to socially distance by emphasizing COVID risks based on COVID deaths or cases. Tools such as those provided by behavioural economics, or other methods such as scare tactics, might better counteract the negative effects of Republican partisanship on adherence to anti-COVID interventions, than increasing their stringency^63^.

## Supplementary Information

Supplementary Information.

## Data Availability

The datasets generated during and/or analysed during the current study are available from the corresponding author on reasonable request.
