# Supplementary material for: Association of Republican partisanship with US citizens’ mobility during the first period of the COVID crisis
Source: Sci Rep. 2022 May 30;12:8994. doi: 10.1038/s41598-022-12790-5 (PMC9149679; doi:10.1038/s41598-022-12790-5)

Association of Republican partisanship with US citizens' mobility during the first period of the COVID crisis

Dr Guillaume Barbalat, MD PhD & Pr Nicolas Franck, MD PhD

Supplementary Materials

Supplementary Materials 1. Political affiliation based on US states' trifecta (2020)

Democratic States (N=16): California, Colorado, Connecticut, Delaware, District of Columbia, Hawaii, Illinois, Maine, Nevada, New Jersey, New Mexico, New York, Oregon, Rhode Island, Virginia, Washington.

Divided States (N=14): Alaska, Kansas, Kentucky, Louisiana, Maryland, Massachusetts, Michigan, Minnesota, Montana, New Hampshire, North Carolina, Pennsylvania, Vermont, Wisconsin.

Republican States (N=21): Alabama, Arizona, Arkansas, Florida, Georgia, Idaho, Indiana, Iowa, Mississippi, Missouri, Nebraska, North Dakota, Ohio, Oklahoma, South Carolina, South Dakota, Tennessee, Texas, Utah, West Virginia, Wyoming.

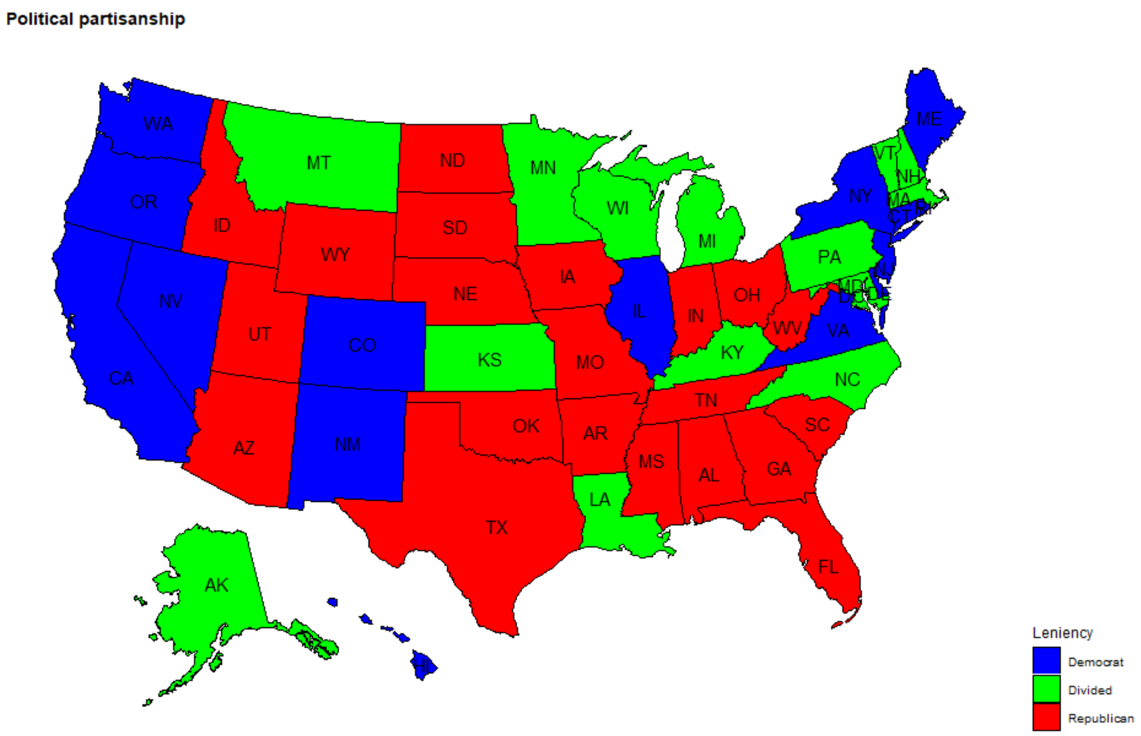

Note that maps were generated using publicly available R package usmap (version 0.5.2) https://usmap.dev.

Supplementary Materials 2. A proposed Directed Acyclic Graph (DAG)

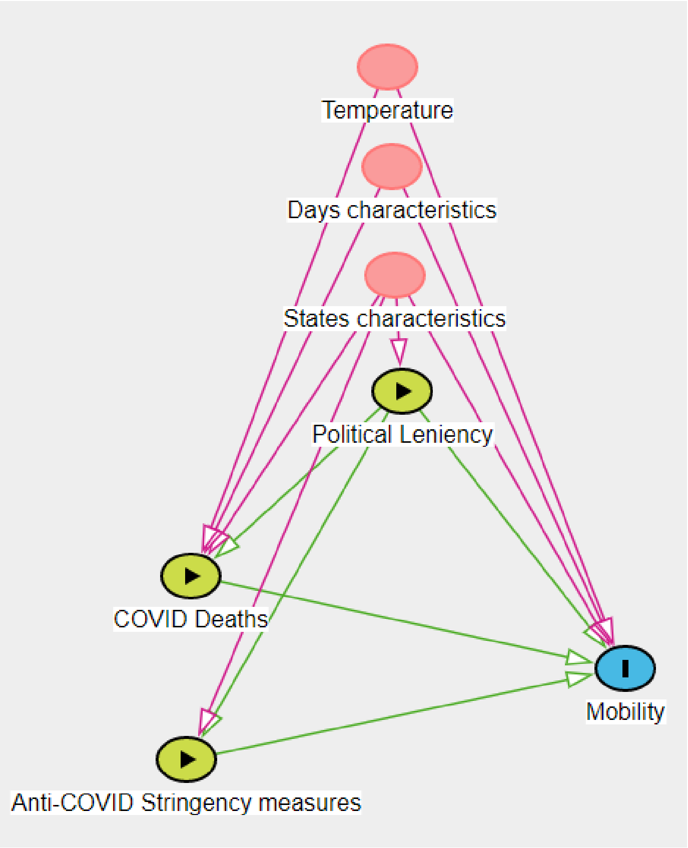

Green nodes represent exposures, Blue nodes represent outcomes, Pink nodes represent parents of exposures and outcomes. Green edges represent causal paths and pink edges represent biasing paths.

Supplementary Materials 3. Geographic distribution of Mobility to transit stations (A & B), anti-COVID stringency measures (C & D), and Number of COVID-related deaths (E & F) at two temporal points (02/15/2010 & 05/31/2020)

A. Mobility to transit stations (% change from baseline)
02/15/2020

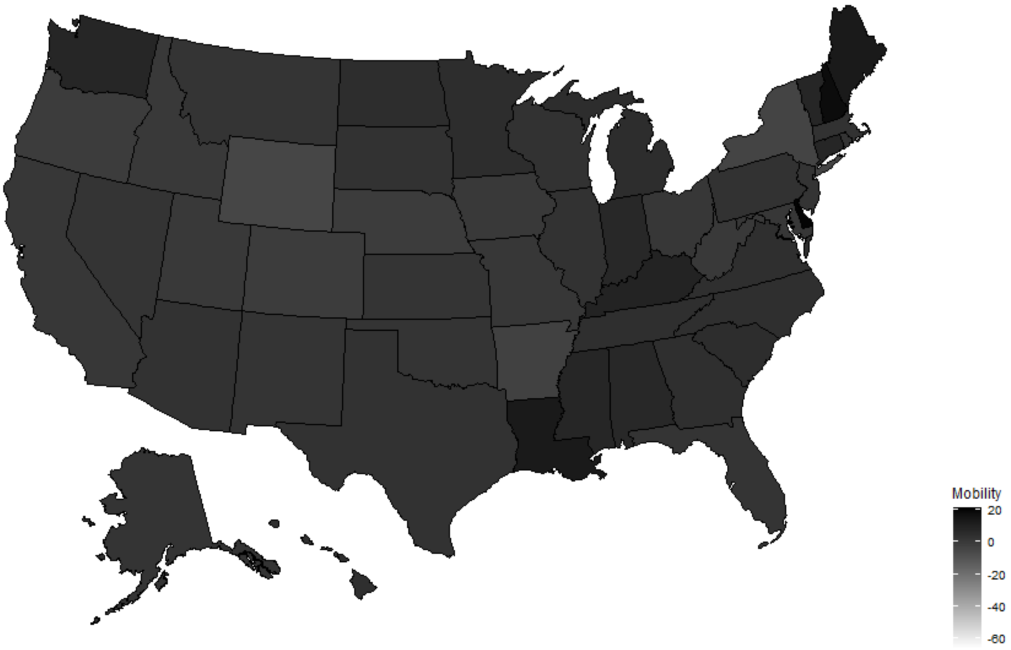

B. Mobility to transit stations (% change from baseline)
05/31/2020

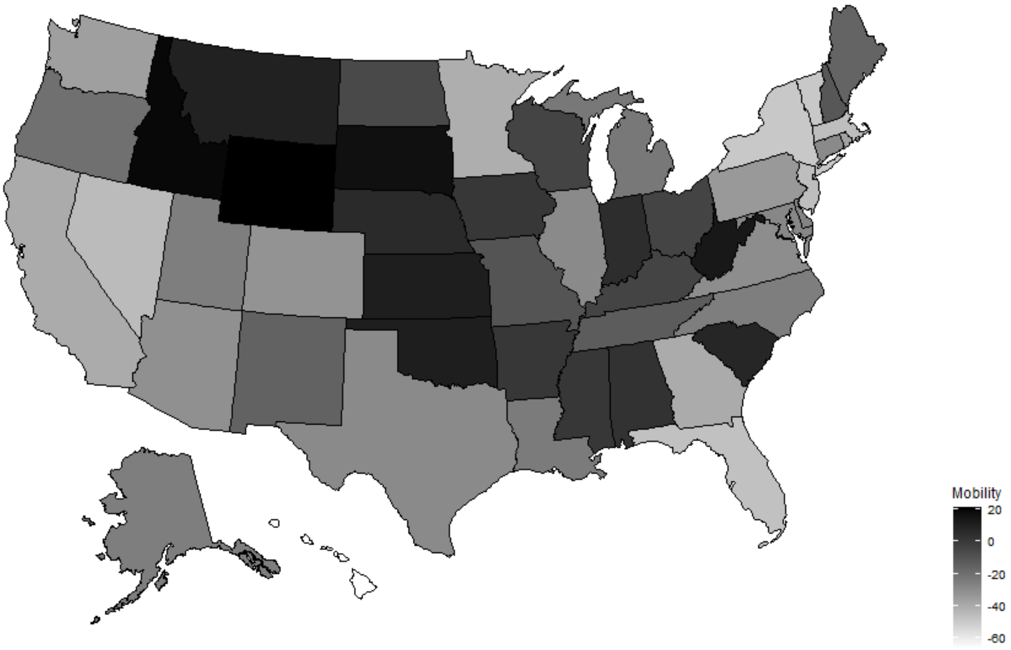

C. anti-COVID Stringency Measures
02/15/2020

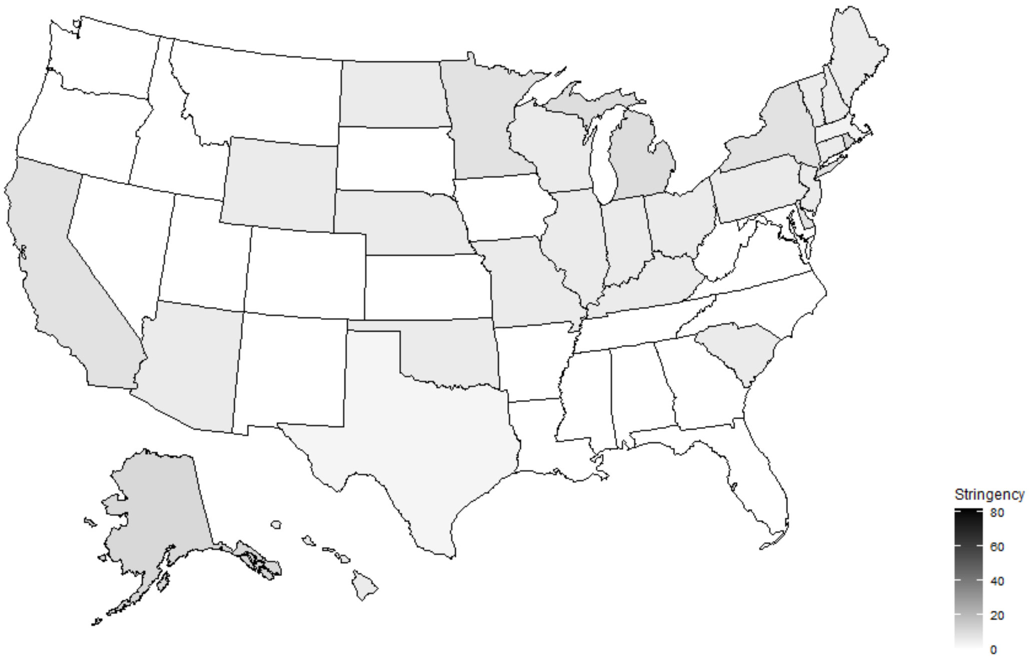

D. anti-COVID Stringency Measures
05/31/2020

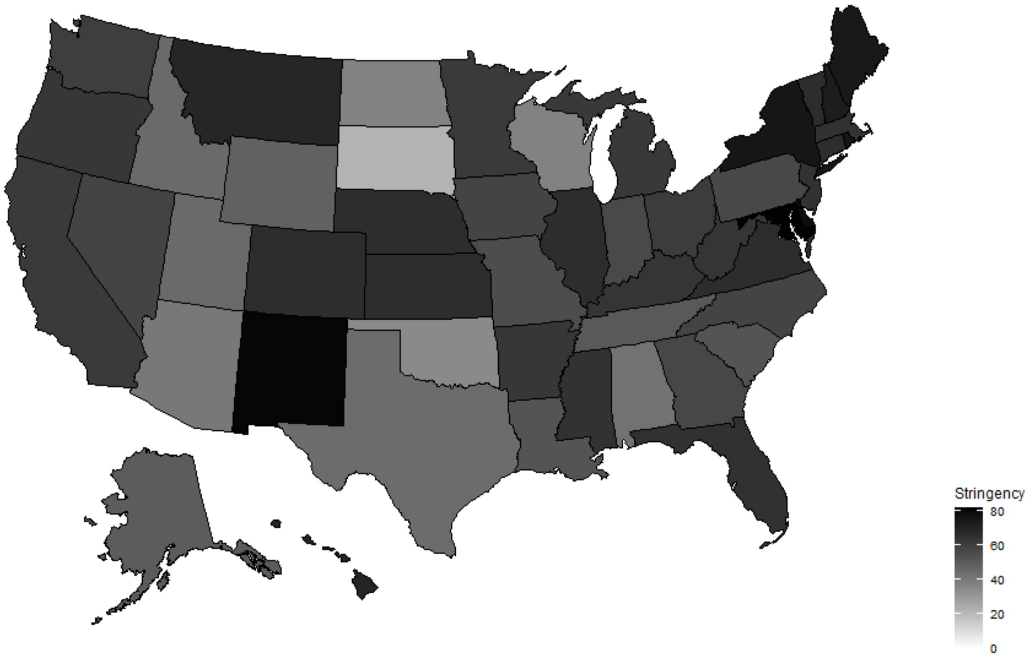

E. cumulative Nb of COVID Deaths
02/15/2020

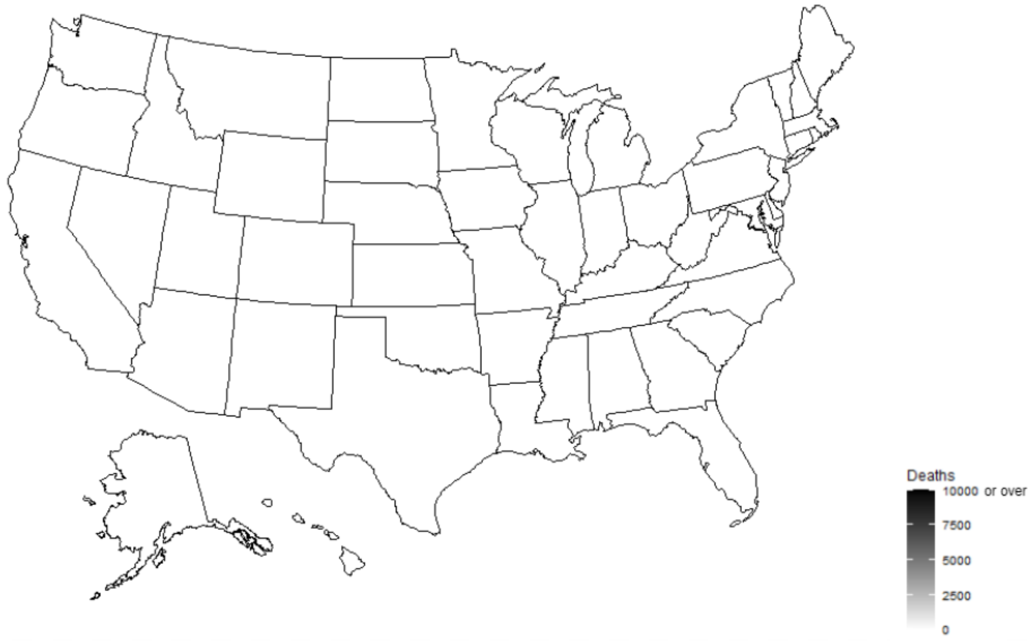

F. cumulative Nb of COVID Deaths
05/31/2020

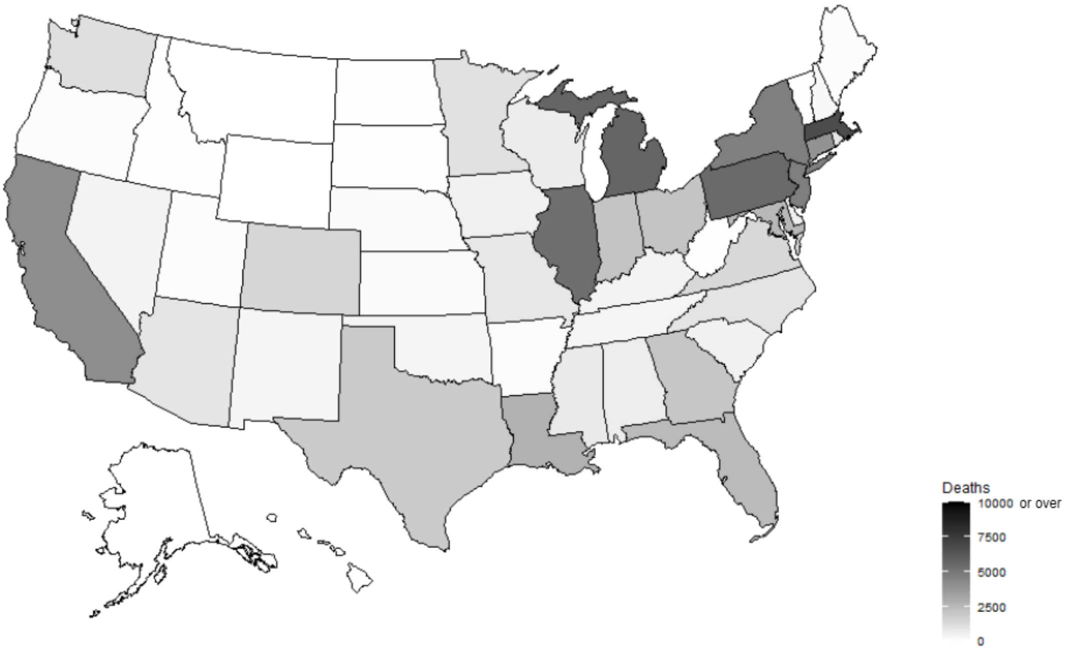

Note that maps were generated using publicly available R package usmap (version 0.5.2) https://usmap.dev.

Supplementary Materials 4. Number of observations in each Stringency and Deaths bin per political group (event-study analysis)

Stringency Bins	Democratic states	Divided states	Republican states
**[0,5.56)**	117	110	229
**[5.56,11.1)**	179	199	235
**[11.1,33.3)**	188	112	249
**[33.3,51.9)**	87	99	365
**[51.9,60.2)**	43	88	367
**[60.2,64.8)**	126	140	219
**[64.8,68.5)**	142	189	237
**[68.5,71.3)**	282	122	94
**[71.3,76.8)**	164	202	164
**[76.8,88]**	384	227	88

Deaths Bins	Democratic states	Divided states	Republican states
**[0,2)**	558	521	826
**[2,9.29)**	99	138	197
**[9.29,25)**	127	125	136
**[25,56)**	89	101	190
**[56,116)**	119	65	214
**[116,233)**	120	115	158
**[233,418)**	132	88	167
**[418,828)**	124	100	168
**[828,2010)**	133	87	170
**[2010,30600]**	211	158	21

Supplementary Materials 5. Sensitivity analysis (Robustness checks)

Results of our sensitivity analyses are provided below. In these analyses, we changed how we measured our outcome variable, how we defined Republican partisanship and perception of COVID risk, and how we accounted for temporality.

- Changes in outcome measurements

The table below shows the mean values, standard errors and p values for the three parameter estimates of interest (Stringency x Republican , COVID Deaths x Republican , Temporal Linear Trend x Republican) for the five other outcome variables provided by the Google COVID-19 Community Mobility Reports database (other than Mobility to transit stations). These were the percent signal change in mobility to: Retail and Recreation places, Workplaces, Groceries and Pharmacies, Residential places, and Parks.

**Retail/Recreation**	**Estimate**	**SE**	**p value**
Stringency x Republican	-0.0013	0.0168	0.9379
COVID Deaths x Republican	-0.0012	0.0003	**0.0007**
Temporal Linear trend x Republican	0.0998	0.0170	**0.0000**
**Workplaces**	**Estimate**	**SE**	**p value**
Stringency x Republican	0.0604	0.0143	**0.0001**
COVID Deaths x Republican	-0.0006	0.0002	**0.0007**
Temporal Linear trend x Republican	0.0033	0.0075	0.6612
**Grocery/Pharma**	**Estimate**	**SE**	**p value**
Stringency x Republican	0.0330	0.0116	**0.0064**
COVID Deaths x Republican	-0.0012	0.0003	**0.0006**

Temporal Linear trend x Republican	0.0312	0.0105	**0.0046**
**Residential**	**Estimate**	**SE**	**p value**
Stringency x Republican	-0.0230	0.0069	**0.0017**
COVID Deaths x Republican	0.0004	0.0001	**0.0000**
Temporal Linear trend x Republican	-0.0090	0.0038	**0.0220**
**Parks**	**Estimate**	**SE**	**p value**
Stringency x Republican	0.0053	0.0619	0.9325
COVID Deaths x Republican	-0.0031	0.0028	0.2712
Temporal Linear trend x Republican	0.1996	0.0908	**0.0326**

The figures below show event-study plots for these five measures.

Mobility to Retail and Recreation places

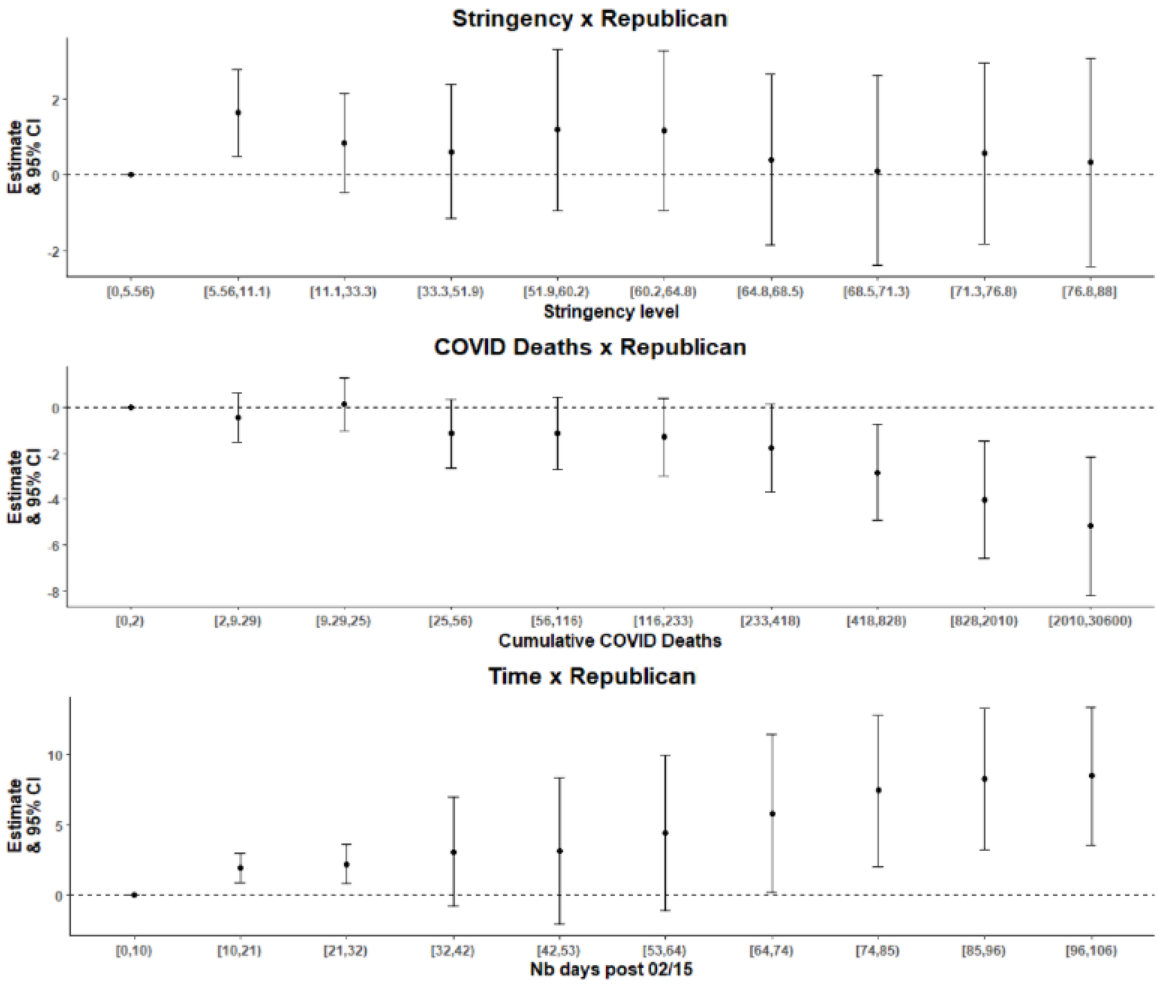

Mobility to Workplaces

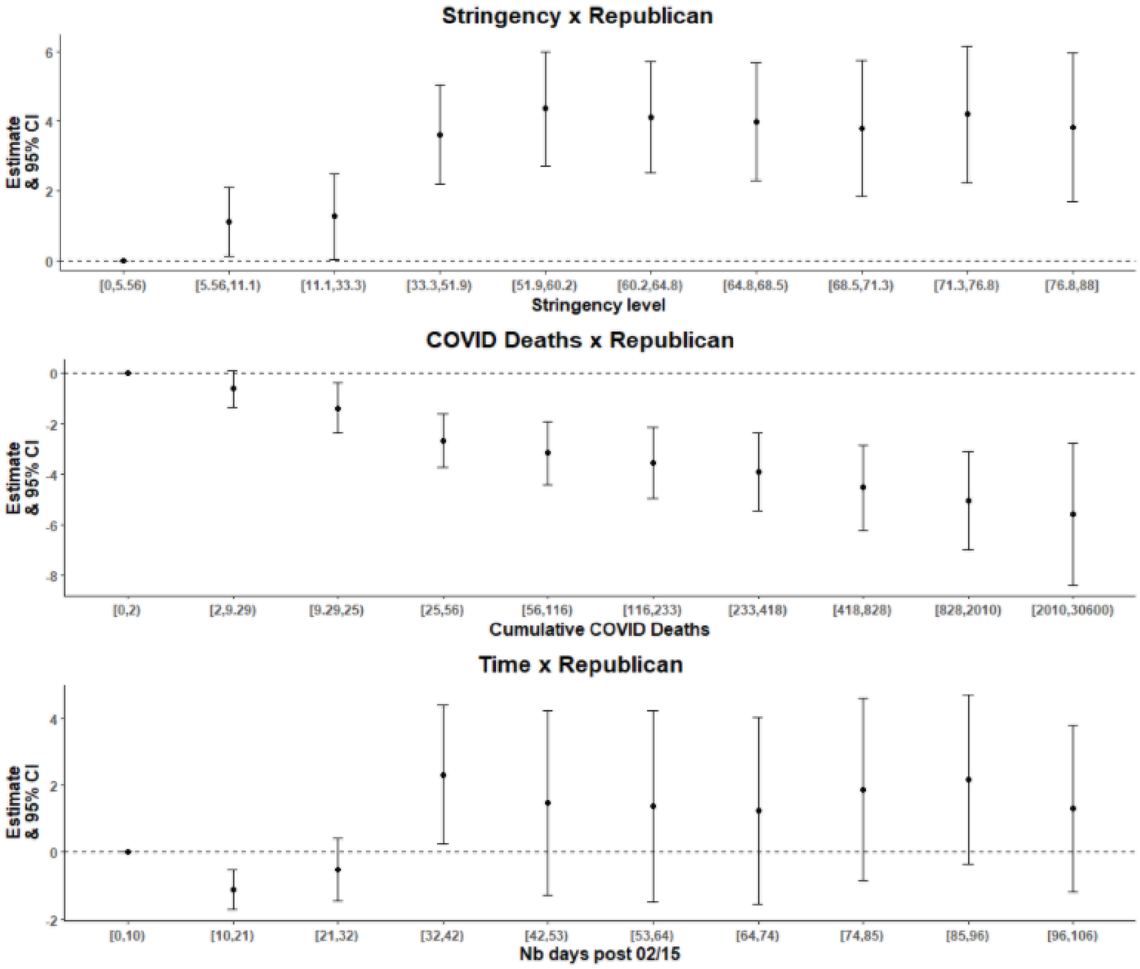

Mobility to Grocery/Pharma

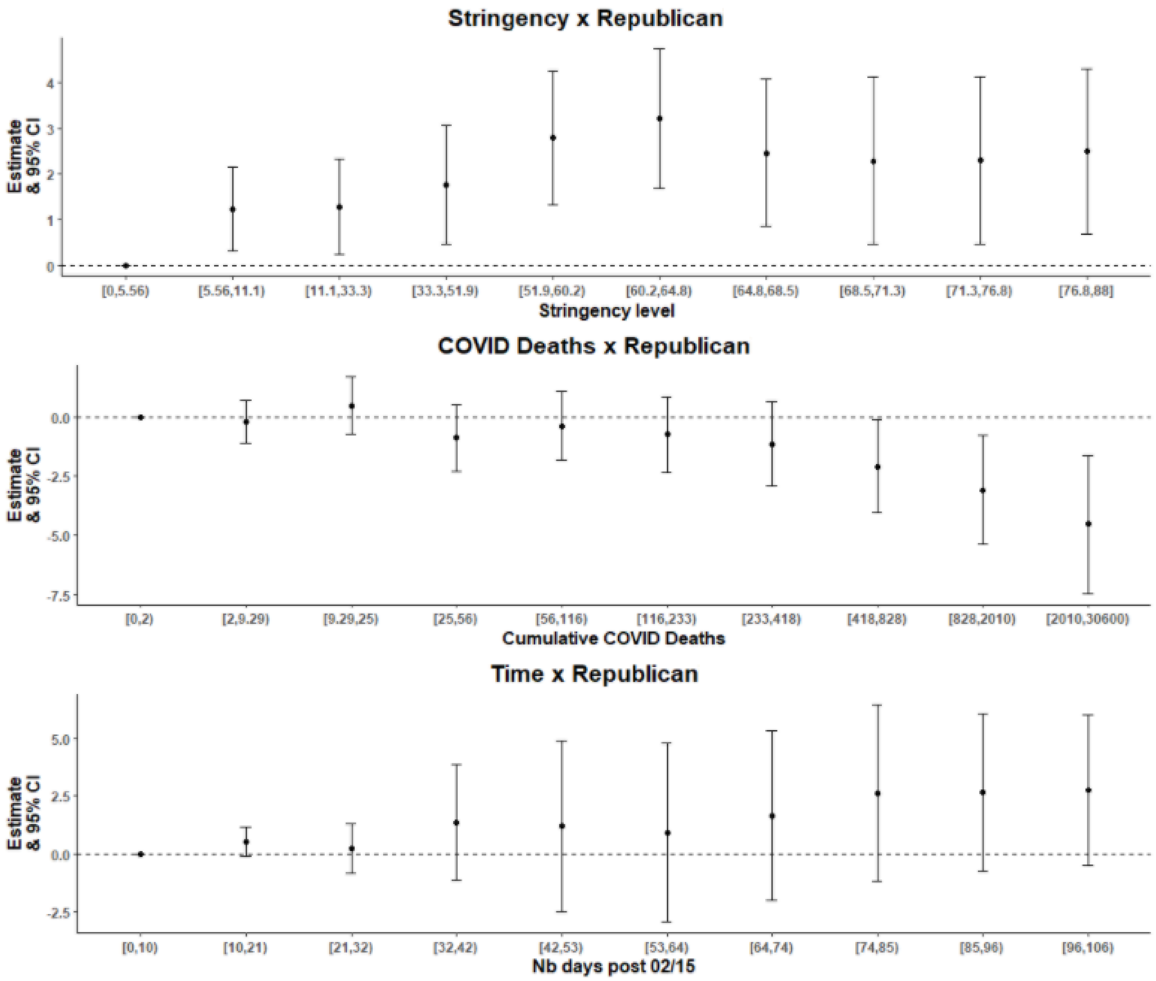

Mobility to Residential places

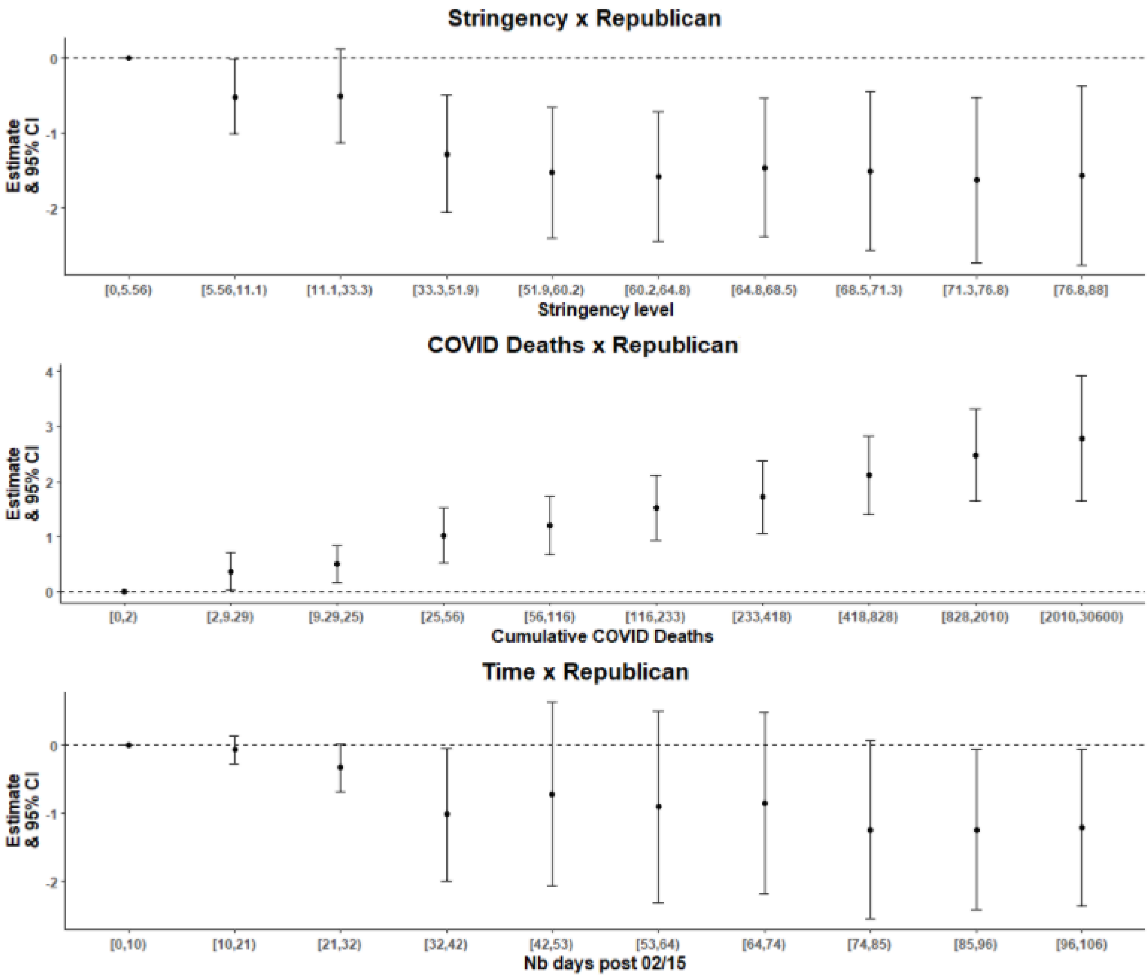

Mobility to Parks

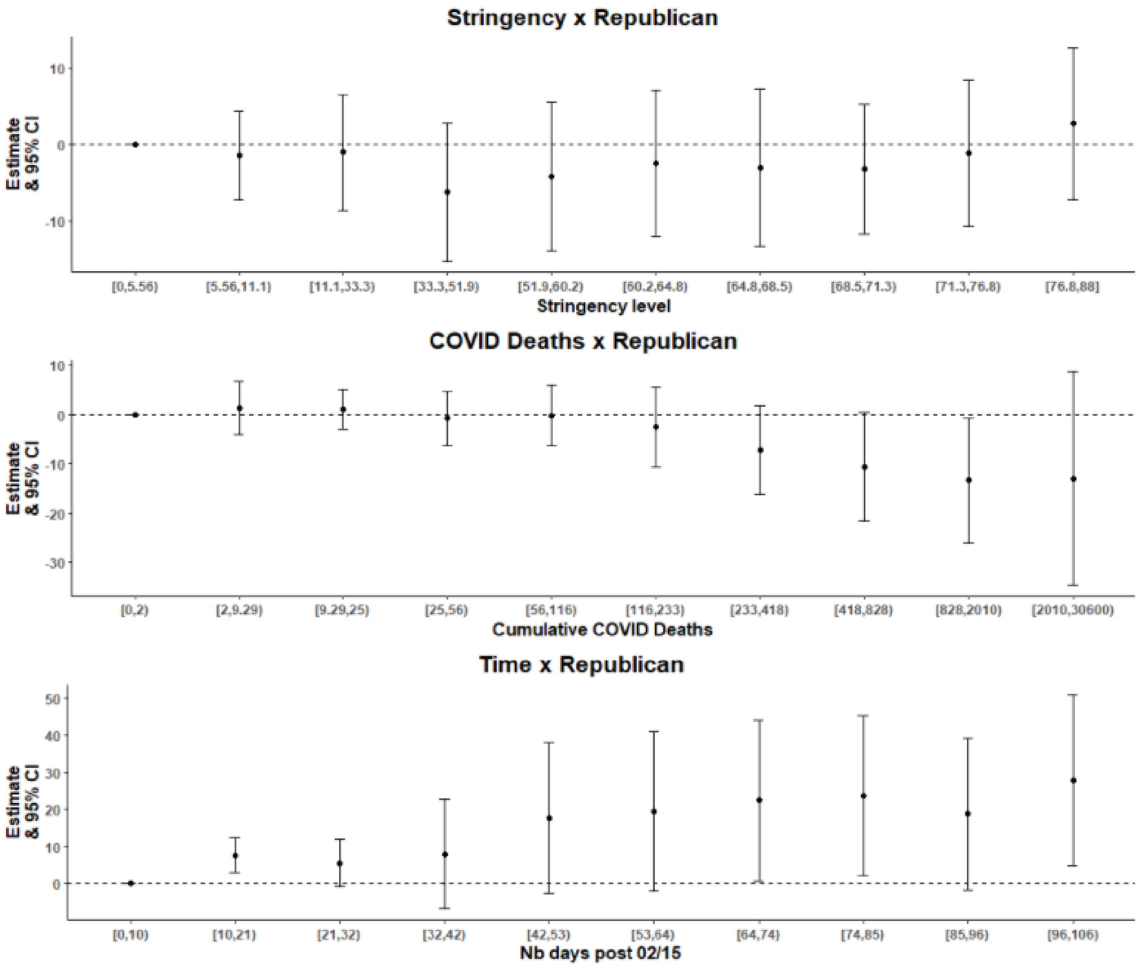

- Changes in measurement of Republican partisanship

The table below shows the mean values, standard errors and p values for the three parameter estimates of interest (Stringency x Republican , COVID Deaths x Republican , Temporal Linear Trend x Republican) using the percentage of voters for the Republican candidate at the 2016 election (Pdt Trump) as an index of Republican partisanship .

	**Estimate**	**SE**	**p value**
**Stringency x Republican**	0.0057	0.0016	**0.0007**
**COVID Deaths x Republican**	-0.0002	0.0000	**0.0001**
**Temporal Linear trend x Republican**	0.0095	0.0012	**0.0000**

- Measurement of perception of COVID risk (using COVID Cases rather than Deaths).

The table below shows the mean values, standard errors and p values for the three parameter estimates of interest (Stringency x Republican , COVID Cases x Republican , Temporal Linear Trend x Republican) using the cumulative number of COVID cases (rather than deaths) as a measure of the perception of COVID risk.

	**Estimate**	**SE**	**p value**
**Stringency x Republican**	0.0721	0.0277	**0.0121**
**COVID Cases x Republican**	-0.0002	0.0000	**0.0000**
**Temporal Linear trend x Republican**	0.1368	0.0214	**0.0000**

- Accounts of temporality

    - Modeling the correlation structure of the error term

The table below shows the mean values, standard errors and p values for the three parameter estimates of interest (Stringency x Republican , COVID Deaths x Republican , Temporal Linear Trend x Republican) modeling the correlation structure of the error term as sequences of non-seasonal Autoregressive Moving Average (ARMA) of daily values for each state, with p=1 lag)

	**Estimate**	**SE**	**p value**
**Stringency x Republican**	0.0664	0.0241	**0.0058**
**COVID Deaths x Republican**	-0.0035	0.0008	**0.0000**
**Temporal Linear trend x Republican**	0.1277	0.0203	**0.0000**

- <u>Increasing the length of the study period (till the end of February 2021)</u>

The figure below shows event study plots over a longer period of analysis (from Feb the 15th 2020 till Feb the 23rd 2021), with Mobility to transit stations as the outcome variable.

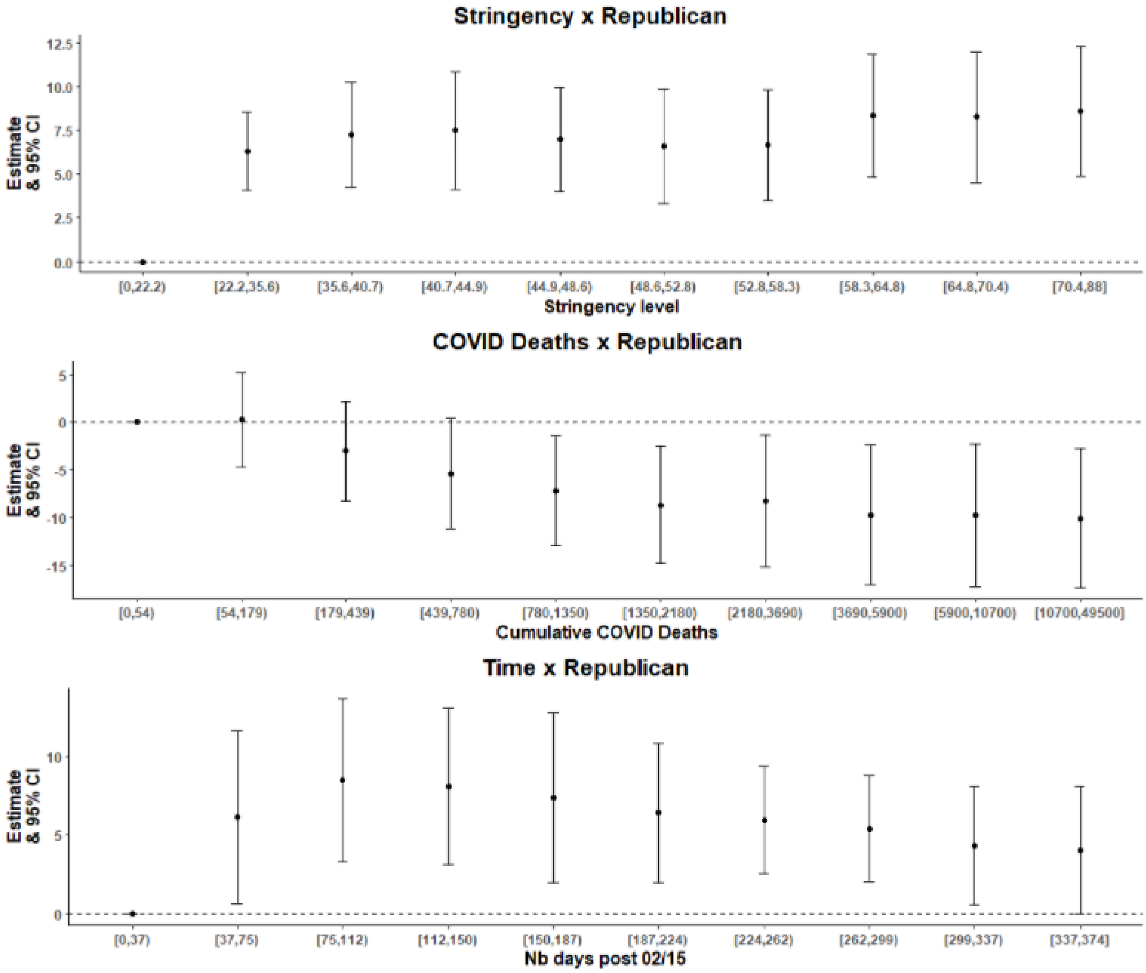

Supplement: Supplementary file 1 — Supplementary Information. [file 41598_2022_12790_MOESM1_ESM.pdf]
